# Genetic and historic evidence for climate-driven population fragmentation in a top cetacean predator: the harbour porpoises in European water

**DOI:** 10.1098/rspb.2010.0412

**Published:** 2010-05-05

**Authors:** Michaël C. Fontaine, Krystal A. Tolley, Johan R. Michaux, Alexei Birkun, Marisa Ferreira, Thierry Jauniaux, Ángela Llavona, Bayram Öztürk, Ayaka A Öztürk, Vincent Ridoux, Emer Rogan, Marina Sequeira, Jean-Marie Bouquegneau, Stuart J. E. Baird

**Affiliations:** 1 MARE Centre—Laboratory for Oceanology, University of Liège, B6c, 4000 Liège, Belgium; 2 INRA, UMR CBGP, Campus International de Baillarguet, CS 30016, 34988 Cedex, France; 3 Marine Mammal Division, Institute of Marine Research, Bergen, Norway; 4 Applied Biodiversity Research, South African National Biodiversity Institute, Private Bag X7, Claremont 7735, Cape Town, South Africa; 5 Génétique des Microorganismes, Département des Sciences de la Vie, Institut de Botanique B22, Université de Liège, 4000 Liège, Belgium; 6 Laboratory of Biotechnological Research in Ecology, Medicine and Aquaculture (BREMA), Simferopol, Ukraine; 7 Portuguese Wildlife Society/CBMA, Universidade do Minho, Thi, 4710-057 Braga, Portugal; 8 Management Unit of the North Sea Mathematical Models, Royal Belgian Institute of Natural Sciences, 100 Gulledelle, 1200 Brussels, Belgium; 9 Department of Pathology, University of Liege, Sart Tilman B43, 4000 Liège, Belgium; 10 Coordinadora para o Estudio dos Mamiferos Mariños, CEMMA, Gondomar, Spain; 11 Faculty of Fisheries, Istanbul University, Ordu Cad. 200, Laleli-Istanbul, Turkey; 12 Centre de Recherche sur les Mammifères Marins, Université de La Rochelle, 17071 La Rochelle Cedex, France; 13 Department of Zoology, Ecology and Plant Science, University College, Cork, Ireland; 14 Instituto da Conservação da Natureza e Biodiversidade, Rua de Santa Marta, 55, 1150-999 Lisboa, Portugal; 15 CIBIO, Campus Agrário de Vairão, R. Monte-Crasto, 4485-661 Vairão, Portugal

**Keywords:** cetacean, climate change, habitat fragmentation, population genetics, coalescence

## Abstract

Recent climate change has triggered profound reorganization in northeast Atlantic ecosystems, with substantial impact on the distribution of marine assemblages from plankton to fishes. However, assessing the repercussions on apex marine predators remains a challenging issue, especially for pelagic species. In this study, we use Bayesian coalescent modelling of microsatellite variation to track the population demographic history of one of the smallest temperate cetaceans, the harbour porpoise (*Phocoena phocoena*) in European waters. Combining genetic inferences with palaeo-oceanographic and historical records provides strong evidence that populations of harbour porpoises have responded markedly to the recent climate-driven reorganization in the eastern North Atlantic food web. This response includes the isolation of porpoises in Iberian waters from those further north only approximately 300 years ago with a predominant northward migration, contemporaneous with the warming trend underway since the ‘Little Ice Age’ period and with the ongoing retreat of cold-water fishes from the Bay of Biscay. The extinction or exodus of harbour porpoises from the Mediterranean Sea (leaving an isolated relict population in the Black Sea) has lacked a coherent explanation. The present results suggest that the fragmentation of harbour distribution range in the Mediterranean Sea was triggered during the warm ‘Mid-Holocene Optimum’ period (approx. 5000 years ago), by the end of the post-glacial nutrient-rich ‘Sapropel’ conditions that prevailed before that time.

## 1. Introduction

Changes in the environment can affect the behaviour and fitness of individual organisms and the viability of populations. Populations can respond to changing habitats by adapting (through natural selection or phenotypic plasticity), moving (to avoid habitat of reduced suitability, or take advantage of emerging habitat), by adjusting population size or some combination of the above. Both natural selection and genetic drift can shape populations as they evolve in this context.

In the North Atlantic, studies have increasingly reported strong shifts in plankton and fish assemblages with the contemporaneous climate and ocean warming (Beaugrand *et al*. 2002, 2008; Richardson & Schoeman 2004; Perry *et al*. 2005). These shifts in marine habitat and community structure are expected to drive major changes in the distribution, density and dispersal of apex predators such as marine mammals, with reduction in density in marginal parts of the habitat leading to fragmentation, local displacement or extinction if the habitat becomes unsuitable (Ryther 1969; Learmonth *et al*. 2006; O'Corry-Crowe 2008; de Bruyn *et al*. 2009; Marx & Uhen 2010). Such evidence is, however, hard to capture for highly mobile marine predators (Kintisch 2006) such as cetacean species (Learmonth *et al*. 2006; O'Corry-Crowe 2008). Key to answering how these apex predators will deal with current and future change in their environment lies in (i) understanding how cetaceans dealt with past climate changes and (ii) determining the impact of changing climate on cetaceans over contemporary time scales (O'Corry-Crowe 2008).

Past shifts in the Earth's climate have led to the alteration or loss of existing environments and the creation of new ones. These changes have been associated with speciation events, adaptive radiations and extinctions, population expansions and contractions, and changes in individual dispersal and breeding behaviour (Hewitt 2000, 2004; Hofreiter & Stewart 2009). Recent studies supported such processes in cetacean species by providing evidence that past changes in the environment, and in particular global change in temperature and diatoms richness that are dominant marine primary producers at the basis of marine food web, were significantly linked to the species diversity of mysticete and odontocete whales (Pastene *et al*. 2007; O'Corry-Crowe 2008; Marx & Uhen 2010). The contemporaneous climate change is however unique with respect to its speed and to the fact that it embeds in global change where both natural and anthropogenic influences act in synergy on ecosystems (Brook *et al*. 2008; Pimm 2008, 2009). Understanding how these factors affect marine systems is therefore a crucial issue.

In this study, we investigated the demographic history of a small coastal cetacean widely distributed in the North Atlantic, the harbour porpoise *Phocoena phocoena*, with regards to recent variation in its habitat. At around 1.5 m in length and 50 kg in weight, it is the smallest cetacean in the North Atlantic (Read 1999), and often considered as a species living in the ‘fast lane’ (Read & Hohn 1995; Lockyer 2007). Reproductive costs of harbour porpoises are high (Lockyer 2007), as females are often gestating and lactating at the same time and parturition occurs shortly before mating (Lockyer 2003). Given their limited capacity to store energy, it is assumed that harbour porpoises must feed frequently without prolonged periods of fasting (Koopman *et al*. 1996, 2002). Some authors suggested that fasting periods exceeding as short as 3 days could affect body condition (Kastelein *et al*. 1997; Lockyer 2007). Thus, temporary shortages in prey availability can negatively impact on the animals and are likely to be responsible for changes in their distribution (Santos & Pierce 2003; Santos *et al*. 2004; Johnston *et al*. 2005; Herr *et al*. 2009). Relatively continuous accessibility to adequate prey is therefore critical. Any changes in prey availability may affect energy stores, and ultimately survival (MacLeod *et al*. 2007). Therefore, we expect the distribution of this species to be strongly tied to variation in the primary and secondary productivity that provides the basis for apex consumers (Lockyer 2007).

**Figure 1.**
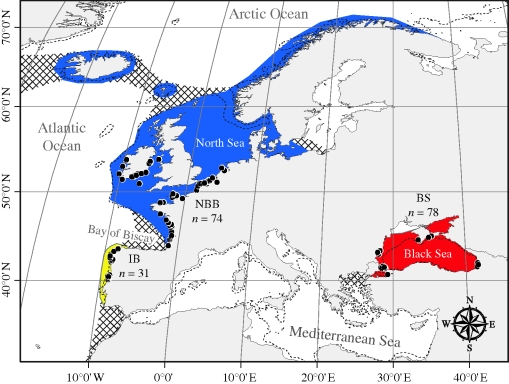
Sampling and distribution range of harbour porpoises in European waters. The filled and hatched surfaces indicate the known and the possible distribution range, respectively. The coloured surfaces show the genetically distinct populations identified in Fontaine *et al*. (2007): the Black Sea porpoises (red, *n* = 78), the Iberian porpoises (yellow, *n* = 31) and the porpoises in northern Bay of Biscay waters (blue, *n* = 78). The dotted line shows the margin of the continental shelf (isobath −200 m) and the black circles the sampling locations.

The harbour porpoise is currently distributed fairly continuously throughout cold to temperate coastal waters of the North Pacific, the North Atlantic and in the Black Sea, but it is absent from the Mediterranean (Read 1999). The southeastern part of the distribution range in the North Atlantic is of particular interest in regard to recent climate change, as it covers the biogeographic transition between arctic/boreal species and subtropical species (Southward *et al*. 1995). Changes in distribution patterns and abundance in this region are thus more evident than elsewhere in Europe for many marine species (Southward *et al*. 1995; Beaugrand *et al*. 2008). In this southeastern part of the North Atlantic, the harbour porpoise displays a disjunct distribution range. The absence of porpoises in the Mediterranean splits the Black Sea range from the Atlantic range. Black Sea porpoises are therefore recognized as a subspecies *P. p. relicta* distinct from the Atlantic one *P. p. phocoena* (Rosel *et al.* 1995), and from an evolutionary perspective, as an evolutionary significant unit (ESU; Moritz 1994, 2002) independent from the Atlantic one (Tolley & Rosel 2006; Viaud-Martinez *et al*. 2007). More recently, in a study covering the entire eastern Atlantic range, Fontaine *et al*. (2007) discovered a second disjunction, a population restricted to the cold-water upwelling zone along the Atlantic coasts of Iberia (IB) (Fiùza 1983) and distinct from porpoises in the northern Bay of Biscay (NBB), which are part of the main North Atlantic range, extending from French waters fairly continuously to the Arctic (figure 1). Fontaine *et al*. (2007) argued that oceanographic conditions, specifically those conditioning food availability, impose strong constraints on the dispersal, and thus the genetic structure of the species. If this hypothesis is correct, the existing genetic structure across the North Atlantic range will have a recent origin, as the oceanographic conditions in the eastern North Atlantic, in the Mediterranean Sea and in the Black Sea have changed dramatically over the course of the Holocene (Alley *et al*. 2003; Mayewski *et al*. 2004; Osborn & Briffa 2006; Springer *et al*. 2008; Rohling *et al*. 2009).

As a cold temperate species, porpoises are unlikely to favour the current conditions of the southern Bay of Biscay and the Mediterranean Sea, with deep, warm and oligotrophic (nutrient-poor) waters (Fontaine *et al*. 2007). However, in the recent past, these waters have at times been cold and nutrient-rich, and it seems likely that porpoise ranges have closely tracked this nutrient availability. This would suggest the Iberian porpoise range could have been continuous with that of North Atlantic waters during recent cold periods that affected European climate (Grove 2004). There have been a number of notable cold periods between the Last Glacial Maximum (LGM, 18 000 years before present (yr BP)) and the most recent cold period, known as the ‘Little Ice Age’ (LIA). The period of continuity between the Black Sea and Atlantic ranges is a much more long standing question (Frantzis *et al*. 2001). The straits of Bosphorus and Dardanelle have been periodically closed during the Quaternary, but have been open continuously since approximately 8400 years ago (Major *et al*. 2002; Rohling *et al*. 2009). Analyses of genetic polymorphism at the non-coding region of the mitochondrial genome (the control region, mtDNA CR) (Tolley & Rosel 2006; Viaud-Martinez *et al*. 2007) suggested, however, a much older splitting time between Atlantic and Black Sea mitochondrial clades (at least 175 000 years ago).

In order to investigate the splitting process among the three porpoise populations identified in the Northeast Atlantic, we extracted historical information from genetic polymorphism data using model-based Bayesian coalescent approaches, and correlated this genetic inference with palaeo-oceanographic and historical records. Specifically, we tested whether the genetic divergence observed among harbour porpoise populations in the Northeast Atlantic is compatible with the recent changes reported in the seascape and marine assemblages, or is more likely owing to ancient vicariance processes.

## 2. Material and methods

### (a) Microsatellite genotypes

Individual genotypes at 10 microsatellite loci were from Fontaine *et al*. (2007). To avoid computation time becoming intractable for the coalescent approach described below, we restricted the analyses to 183 harbour porpoises at the borders of the genetic barriers evidenced in Fontaine *et al*. (2007): 74 animals from the northern Bay of Biscay, 31 collected along the Iberian coast and 78 from the Black Sea (figure 1). Details regarding descriptive statistics on the microsatellite dataset can be found in Fontaine *et al*. (2007).

### (b) Data analysis

We analysed the datasets using the ‘isolation with migration’ (IM) model of population divergence (Hey & Nielsen 2004). The model uses coalescent simulations within a Bayesian inference framework to estimate marginal probability distributions for six demographic parameters scaled by the mutation rate *μ*: the time since population-splitting (*T* = *t *μ**), measures of neutral population genetic diversity of the two current and one ancestral population (*θ*_1_, *θ*_2_, *θ*_A_) proportional to their effective population sizes *N*_e_ (*θ* = 4*N*_*e*_ *μ*) and bidirectional migration rates (*M*_1_ = *m*_1_/*μ*, *M*_2_ = *m*_2_/*μ*) (Hey & Nielsen 2004).

We analysed population-splitting first comparing the IB with the NBB population. Then, we compared the two ESUs, i.e. harbour porpoises from the Black Sea (BS) with those in the Atlantic (AT, including IB and NBB). This procedure was adopted because previous works suggested that the Black Sea harbour porpoises separated from Atlantic populations long before IB porpoises separated from the NBB populations. This is supported by complete lineage sorting of mtDNA haplotypes between porpoises from the Black Sea and the North Atlantic (Tolley & Rosel 2006; Viaud-Martinez *et al*. 2007), in contrast with the lack of private mitochondrial and microsatellite alleles between the Iberian and northern Bay of Biscay (Tolley & Rosel 2006; Fontaine *et al*. 2007; Viaud-Martinez *et al*. 2007). Pooling Atlantic populations may induce some bias in our inference regarding the Black Sea–Atlantic comparison, because the model assumes panmixia within each population. However, a recent simulation study showed that the model estimates were robust to such departure (Strasburg & Rieseberg 2010). On the other hand, comparing each pair of populations separately (IB–BS and NBB–BS), thus ignoring a population in the Atlantic (IB or NATL) that is more closely related to the other, profoundly violates the basic assumptions of the model (Hey & Nielsen 2004) and could have a more profound impact on the model estimates (Strasburg & Rieseberg 2010).

We conducted Markov Chain Monte Carlo (MCMC) simulations using the IM program (Hey & Nielsen 2004), assuming a stepwise mutation model of microsatellite evolution and uniform prior distributions over parameter ranges. Prior boundaries were empirically determined to ensure that the posterior distributions fell completely within the prior range. The modes of the posterior probability distributions were thus taken as the maximum-likelihood estimate (MLE) of the parameters. We estimated the credibility intervals as the 90 per cent highest probability density intervals (HPDIs) (i.e. the shortest span that includes 90% of the probability density of a parameter).

Convergence of such a kind of analysis and dataset is a tricky issue. To ensure reliable convergence towards the stationary distribution, we monitored multiple independent runs for the two datasets, each with 50–100 independent heated chains under Metropolis coupling to improve mixing (Hey & Nielsen 2004). Each run was initiated with a burn-in period of at least 300 000 updates, with each multi-chain simulation lasting several months (the longest runs were up to 3.1 × 10^7^ iterations, see the electronic supplementary material, table S1, for details regarding runs). Mixing properties of the MCMC were assessed by visual inspection of the parameter trend plots and by examining the level of autocorrelation between initial and final parameter values (using the effective sample size, ESS). Analyses were considered to have converged upon the stationary distribution if independent runs generated similar posterior distributions, with each having a lowest ESS of 50 for each estimated parameter as recommended in Hey & Nielsen (2004).

To convert posterior estimates of the model scaled by the mutation rate *μ* (i.e. *T* and *θ*) into demographic units (i.e. *t* and *N*_e_), we used a value of *μ* = 5.0 × 10^−4^ mutation per generation, which is considered as the average mutation rate over many species (Brinkmann *et al*. 1998; Estoup & Angers 1998; Ellegren 2000; Estoup *et al*. 2002; Sun *et al*. 2009). Assuming a generation time (*G*) for harbour porpoise ranging between 5 (*G*_L_, the average age of females at first parturition) (Lockyer 2003) and 7 years (*G*_H_, as suggested by Tolley & Rosel 2006), population-splitting time (*T*) can be converted to calendar years (*t*) and estimates of population mutation rates (*θ*_1_, *θ*_2_ and *θ*_A_) can be converted into estimates of effective population size parameters (*N*_1_, *N*_2_ and *N*_A_, respectively, in a number of individuals) as described in Hey & Nielsen (2004). Migration parameters in the model (*M*_1_ and *M*_2_) can be used to obtain population migration rates (2*N*_1_*m*_1_ and 2*N*_2_*m*_2_, i.e. the effective number of migrants per generation into population 1 and 2, respectively) using the MLE estimates of current *θ* and of *M* (2*N*_1_*m*_1_ = *θ*_1_*M*_1_/2 and 2*N*_2_*m*_2_= *θ*_2_*M*_2_/2) (Hey & Nielsen 2004).

## 3. Results and discussion

### (a) Convergence of IM runs

**Figure 2.**
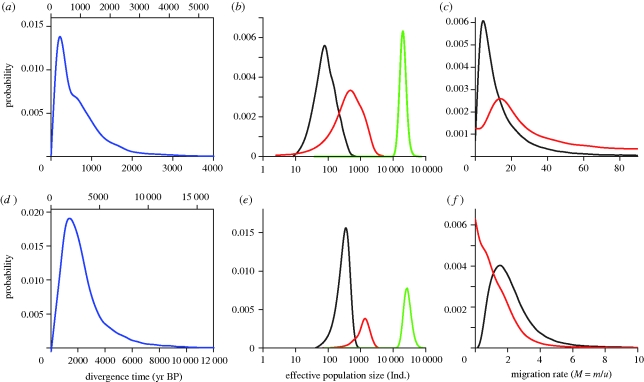
Marginal posterior probability densities for each parameter of the IM model. Curves are shown for the analyses comparing harbour porpoise populations from (*a*–*c*) Iberian (IB) versus northern Bay of Biscay (NBB), and (*d*–*f*) the Black Sea (BS) versus Atlantic (AT). The bottom and top *x*-axes of plots (*a*,*d*) show the time scales converted into calendar years before present (yr BP) using two generation times 5 and 7 years (bottom and top *x*-axes, respectively). (*b*) Black line, IB; red line, NBB; green line, ancestral. (*c*) Migration rate into IB, black line; migration rate into NBB, red line. (*e*) Black line, BS; red line, AT; green line, ancestral. (*f*) Migration rate into BS, black line; migration rate into AT, red line.

After several steps of trimming, the replicated IM simulations displayed good mixing properties of the coupled MCMCs as suggested by the parameter trend line plots (data not shown) with an ESS of at least 71 for the IB–NBB analyses and 53 for AT–BS analyses (details on these runs are provided in the electronic supplementary material, table S1). IM replicates provided consistent similar posterior probability densities for all parameters (electronic supplementary material, figures S1 and S2). All these indications suggested that we have sufficiently explored the parameter space. We selected runs that best converged (i.e. those displaying the highest ESS estimates) to convert them into demographic units (figure 2 and table 1) and to interpret the outputs.

**Table 1.** Maximum-likelihood estimates and 90% HPDIs (between brackets) of parameter estimates under the IM model. (2*N*_1_*m*_1_ and 2*N*_2_*m*_2_ are the effective number of migrants per generation, respectively, into population 1 and 2. 1, population 1; 2, population 2; A, ancestral population.)

parameter	Iberian (1) × northern (2) Bay of Biscay	Black Sea (1) × Atlantic (2)
*t* (years)^a^	175–245 (55–2079)	1325–1855 (125–6,335)
*N*_1_	79 (22–328)	362 (148–573)
*N*_2_	353 (148–2243)	1393 (543–2453)
*N*_A_	19 688 (13 313–30 113)	26 925 (16 875–45 225)
2*N*_1_*m*_1_ = *θ*_1_*m*_1_/2	0.34	0.54
2*N*_2_*m*_2_ = *θ*_2_*m*_2_/2	4.65	0

^a^Splitting time estimates (*T = t *μ**) were converted into calendar years using a per-generation mutation rate (*μ*) of 5.0 × 10^−4^ and two generation times (*G*) 5 and 7 years.

### (b) Iberian versus northern Bay of Biscay

Results from the IM model for the isolation of IB porpoises from those of the NBB are shown in figure 2*a*–*c* and summarized in table 1. Estimated posterior probability distributions of effective population sizes (figure 2*b* and table 1) show that a large ancestral population, with a modal effective size (*N*_A_) of *ca* 20 000 individuals (HPDI: 13 313–30 113), split into two smaller populations: a very small population off the Iberian Atlantic coasts with an effective size of *ca* 80 individuals (HPDI: 22–328), and a larger population in the northern Bay of Biscay with an effective size of *ca* 350 individuals (HPDI: 148–2243) (table 1). The splitting time was clearly resolved, with a sharply peaked posterior distribution (figure 2*a*) and a MLE between 175 and 245 yr BP (HPDI: 55–2079), depending on the generation time used (5 or 7 years). Migration rate distributions (figure 2*c*) show a clear signal of migration in both directions with, however, 14 times higher gene flow from Iberia towards the northern Bay of Biscay after population-splitting (table 1).

The genetic inference thus indicates the barrier to gene flow located in the southern Bay of Biscay (Fontaine *et al*. 2007) rose during the past millennium and most probably during the few last centuries. It is now widely recognized that worldwide anomalies in climatic and oceanic conditions occurred during the past 2000 years (Osborn & Briffa 2006). The most significant according to Osborn & Briffa (2006) include the ‘Medieval Warm Period’ (1200–800 yr BP), the ‘LIA’ (600–150 yr BP) and the modern warm period. During the LIA that culminated 300 years ago, dramatic cooling in oceanographic and climatic conditions in the North Atlantic, and over Europe in particular, had a substantial impact on human activities, for which there are historical records, but also more generally on terrestrial and marine ecosystems (Grove 2004). Fishery records in Europe, which date back as far as the tenth century for some species (Alheit & Hagen 1997), report both significant increases in capture of cold-water fishes (e.g. herring *Clupea harengus*) as far south as in the Bay of Biscay and the southward retreat of warm-water species (e.g. sardine) during the LIA (Alheit & Hagen 1997). Given that cold-water fishes, like herring or sandeel (*Ammoditidae sp*.), are a major component of porpoise diet throughout its current range (Read 1999; Santos & Pierce 2003), it is highly probable that the harbour porpoise's past range was likewise extended southward into the Bay of Biscay. The IM demographic estimates in terms of timing, geneflow and the relative size of the subranges are consistent with such a scenario where harbour porpoises north and south of the Bay of Biscay formed a single range during the LIA cold conditions, and began to fragment with the onset of the current warm period 200–300 yr BP (Osborn & Briffa 2006), together with substantial shifts in the marine community in the eastern North Atlantic and especially in the Bay of Biscay (Poulard & Blanchard 2005). The relatively cold nutrient-enriched conditions generated by coastal upwelling along the Iberian Atlantic coasts (Fiùza 1983) have allowed a small porpoise population and other cold temperate species to persist in these waters up to now (OSPAR Commission 2000). The predominant northward migration signal detected in the genetic data supports a northward exodus of Iberian porpoises during their isolation, as expected for a Northern Hemisphere temperate species in response to ocean warming.

### (c) Atlantic versus Black Sea

Results from the IM model for the isolation of Black Sea porpoises from those in the Atlantic are shown in figures 2*d*–*f* and summarized in table 1. The estimates suggest a very large ancestral effective population size (*N*_A_) of *ca* 30 000 individuals (HPDI: 16 875–45 225). According to the model, this ancestral population split into much smaller populations (*ca* 360 (HPDI: 148–573) individuals in the Black Sea and *ca* 1400 (HPDI: 543–2453) individuals in the Atlantic). The population split is estimated to have occurred 1325–1855 years ago (HPDI: 125–6335, figure 2*d*), depending on the generation time used. Migration rate posterior distributions (figure 2*f*) show a clear signal of migration, though very low, from the Atlantic to the Black Sea, but no evidence of gene flow in the reverse direction (table 1 and figure 2*f*).

Previous studies suggested a much older separation time of Black Sea from Atlantic porpoises based on the analysis of genetic polymorphism at the non-coding region of the mtDNA CR (Tolley & Rosel 2006; Viaud-Martinez *et al*. 2007). We ran further IM simulations using published and new mtDNA sequences (see electronic supplementary material, methods, table S1, S2 and figure S3). Combined microsatellite and mtDNA data did not change inference compared with the results described above, but results from the mtDNA locus alone showed a strong discrepancy with the nuclear loci (electronic supplementary material, figure S3; Viaud-Martinez *et al*. 2007). The Atlantic–Black Sea splitting time estimate based only on the mtDNA CR and converted into calendar years using the formerly admitted range of mutation rates (between 3.3 and 4.3 × 10^−8^ substitutions per site and per year (s.s.yr^−1^) (Tolley & Rosel 2006; Viaud-Martinez *et al*. 2007) was up to three orders of magnitude greater than at nuclear loci. However, any estimate of divergence time is conditioned on the uncertainty regarding the mutation rate estimates, which is very difficult to assess and highly variable among species (Nabholz *et al*. 2008*a*). The IM algorithm accounts for variation in the mutation rate among loci by estimating the relative locus-specific mutation rate as a mutation rate scalar (Hey & Nielsen 2004). Provided we have a sound mutation rate estimate for at least one locus allows us to deduce the mutation rate of the others. Assuming that the average mutation rate for microsatellite loci is 1 × 10^−4^ mutations per year (considering a generation time of 5 years), the mtDNA CR substitution rate can be estimated from our combined analysis at 1.3 × 10^−6^ s.s.yr^−1^ (HPDI: 8.4 × 10^−7^–2.3 × 10^−6^). This is at least two orders of magnitude faster than ‘traditional’ substitution rate estimates derived from interspecific phylogenetic datasets or the fossil record (Hoelzel *et al*. 1991; Henn *et al*. 2009) but, within the intervals of those found with the mean human mtDNA CR pedigree rate estimate derived from a meta-analysis (9.5 × 10^−7^ s.s.yr^−1^) (Howell *et al*. 2003), with ancient DNA datasets in Adélie penguin *Pygoscelis adeliae* (9.6 × 10^−7^ s.s.yr^−1^) (Lambert *et al*. 2002), in Southern elephant seal *Mirounga leonina* 9.8 × 10^−7^ s.s.yr^−1^ HPDI: 1.7 × 10^−9^–2.1 × 10^−6^) (de Bruyn *et al*. 2009) and in Steller sea lions *Eumetopias jubatus* 2.7 10^−7^ s.s.yr^−1^ (Phillips *et al*. 2009). Such a high intraspecific estimate is reminiscent to the time dependency of mtDNA mutation rates recently recognized as a general phenomenon applying to many species (e.g. primates, marine mammals, birds and fishes) (Lambert *et al*. 2002; Howell *et al*. 2003; Ho *et al*. 2005, 2007; Bandelt 2008; de Bruyn *et al*. 2009; Endicott *et al*. 2009; Henn *et al*. 2009). Higher mutation rate estimates are obtained from events calibrated with more recent dates, such as pedigrees, and lower estimates result when events are calibrated with older dates.

The anomalous behaviour of the mitochondrion did not only concern the substitution rate, but also estimates of ancestral population genetic diversity (*θ*) from mtDNA CR alone. mtDNA *θ* estimates showed a diametrically opposed pattern to that of nuclear microsatellite loci, with estimates of ancestral population genetic diversity being much smaller than the current population diversity (electronic supplementary material, figure S3 and fig. 4 in Viaud-Martinez *et al*. (2007)). Under neutral evolution of the genetic markers used, historical variation in population size should affect genetic polymorphism at both nuclear and mitochondrial loci in a similar manner (albeit with larger drift effects on the mitochondrion). The opposing pattern we observed here could be the outcome of the large stochastic variance in the coalescence process affecting a single locus (Hey & Machado 2003), but it is worthwhile considering other potential causal factors. First, selective processes may have acted on the mtDNA genome (e.g. selective sweeps). Given the central role of mitochondria in heat and energy production, and the strong energetic constraints faced by porpoises which are small, warm-blooded marine carnivores with a demanding reproductive schedule, detecting a signal of selection on the mitochondrion would seem hardly surprising. Second, heterogeneous mutation rates along the mtDNA CR are poorly captured by the substitution model. Mutational hotspots resulting in high levels of homoplasy could provide a false signal of population expansion (Pakendorf & Stoneking 2005; Galtier *et al*. 2006; Strasburg & Rieseberg 2010). Finally, the discrepancy may be the result of complex and multiple determinants of genetic diversity on the mtDNA genome. A recent study examining mtDNA evolution in mammals showed that mitochondrial rates of evolution generally do not depart from neutral expectations, although they are highly stochastic among species, and essentially unpredictable from knowledge of species biology (Nabholz *et al*. 2008*b*; Galtier *et al*. 2009).

As harbour porpoises currently inhabit productive coastal waters of the North Atlantic and Black Sea (Read 1999), we expected that porpoises were present in the Mediterranean at a time when palaeoceanographic conditions were more productive than the present-day oligotrophic conditions. Geological records indicate that such conditions prevailed in the Mediterranean Sea throughout the deglaciation period following the LGM (approx. 18 000 yr BP) until the Mid-Holocene warm period (approx. 6000 yr BP) (Kerwin *et al*. 1999; Jimenez-Espejo *et al*. 2007; Rohling *et al*. 2009). The strongest contrast with current conditions was in the eastern Mediterranean basin, where organic-rich sediments, termed ‘Sapropel’, accumulated under strong hydrological changes generated by intense fresh water discharges and substantially enhanced primary productivity (Calvert *et al*. 1992; Rohling *et al*. 2009). At the end of this Sapropel episode, approximately 5500 years ago, primary productivity abruptly decreased toward present-day conditions. Demographic estimates from the IM model based on microsatellite data or combined microsatellite and mtDNA data are consistent with porpoise range fragmentation in the Mediterranean starting around 5500 years ago at the end of this Sapropel event, while productive conditions persisted in the northern Aegean Sea and the Black Sea. The posterior estimates of Atlantic–Black Sea time of separation based on microsatellite data firmly place this isolation event in the post-LGM period and point to the post-Sapropel period. The signal of migration from the Atlantic to the Black Sea captured in the IM analysis (figure 2*f* and table 1) probably reflects migration events during the early stage of the divergence process and is consistent with the proposal that the last remnants of Mediterranean porpoises (the ‘admixed’ link between Atlantic and Black Sea) ended up retreating to the Black Sea.

Post-glacial and very recent climate-driven environmental changes have triggered deep reorganization in Northeast Atlantic ecosystems, with changes detected in plankton and fish communities. Our study provides strong evidences of such a response for a small coastal cetacean that has to meet a particularly demanding energetic budget to survive. Harbour porpoise populations in the Northeast Atlantic waters have undergone recent dramatic changes in connectivity and distribution probably related to climate-driven variation in habitat suitability. Our inferences naturally depend upon a specific model of population evolution (IM model), which necessarily simplifies the population demographic history, and on a specific mutation rate. Departures from the model, such as departure from panmixia within population or change in the effective size of diverging populations, may induce some bias in our estimates. However, the model appeared quite robust to these departures (Strasburg & Rieseberg 2010). The biological congruence between our genetic inferences and historical and palaeoceanographic records suggests the model captures the essential aspects of this fragmentation process.

It seems inevitable that marine predators will need to adapt to a changing spatial distribution of primary and secondary production within pelagic marine ecosystems as they did in the past (Marx & Uhen 2010). This reaction to climate change could however be exacerbated on a much shorter time scale by the depletion of major commercial fish stocks induced by intense commercial fisheries (Worm *et al*. 2006, 2009) and/or by detrimental interactions with human activities such as incidental catches, which probably constitute a much more serious short-term threat for small coastal cetaceans such as harbour porpoises (Stenson 2003; Hovelsrud *et al*. 2008). The combination of these effects raises serious concerns regarding the ability of top predators to adapt to such rapidly changing habitat conditions. Addressing these concerns will be a particular challenge for future research and management policy.
